# A qualitative study on coping behaviors and influencing factors among mothers in Japan raising children under three years old while experiencing physical and mental subjective symptoms

**DOI:** 10.1186/s12905-017-0494-8

**Published:** 2018-01-10

**Authors:** Misato Kaso, Kikuko Miyazaki, Takeo Nakayama

**Affiliations:** Department of Health Informatics, School of Public Health, Kyoto University, Yoshida Konoe-cho, Sakyo-ku, Kyoto, 606-8501 Japan

**Keywords:** Mothers, Child-rearing, Coping behaviors, Qualitative research

## Abstract

**Background:**

No studies illustrating the coping behaviors of mothers experiencing physical and mental subjective symptoms, or the factors that contribute to these behaviors, have been investigated. Therefore, the present study sought to develop a conceptual framework on the coping behaviors and contributing factors of mothers experiencing physical and mental subjective symptoms.

**Methods:**

This qualitative study involved theoretical sampling and semi-structured interviews of mothers who were raising children under 3 years of age in Japan and had experienced physical and mental subjective symptoms since giving birth. Women who were pregnant, required regular medical exams, or had difficulty communicating in Japanese were excluded. All mothers were recruited via personal contacts, snowball sampling, and posters at a community center and nursery schools. Analysis was conducted using the constant comparative method. The interview data were extracted in contextual units based on analytical themes, and concepts were generated. Relationships between concepts were investigated and categorized. To confirm theoretical saturation and ensure the validity of the data, a study supervisor was appointed, four qualitative researchers examined the results, and the interview respondents underwent member checking.

**Results:**

There were a total of 21 participants. Thirteen categories were created from 29 concepts identified from the analytical theme *“What do mothers do when raising children under 3 years of age while experiencing physical and mental subjective symptoms?*” While experiencing subjective symptoms, mothers raising children under 3 years of age tended to lead a child-centric lifestyle and were hesitant to visit the doctor, not only because of typical reasons such as time and costs, but also because of factors related to their child. Some circumstances occurring while experiencing physical and mental subjective symptoms led mothers to put their own needs first and attempt to cope on their own as much as possible. As a result, most mothers would only visit a doctor after becoming seriously ill.

**Conclusion:**

Mothers raising children under 3 years of age in Japan tend to put their own needs on hold when experiencing subjective symptoms. As a result, they attempt to cope on their own and, at times, only visit a doctor after becoming seriously ill.

**Electronic supplementary material:**

The online version of this article (10.1186/s12905-017-0494-8) contains supplementary material, which is available to authorized users.

## Background

A survey of 74,000 people by Japan’s Ministry of Health, Labor and Welfare (MHLW) found that the rate of physical and mental symptoms was 1.3 to 1.5 times higher in women of the child-rearing to menopause generation compared with men of the same age span, whereas it was almost equal for men and women in all other age groups [1]. This finding suggests that women of ages spanning from child-rearing to menopause are more susceptible to subjective symptoms. Furthermore, looking at child status, women who are raising children are at a greater risk of mental health symptoms such as irritability and psychological disturbances [2–4], as well as worse self-rated health and greater fatigue [5] compared with women who are not. In addition a MHLW longitudinal survey of 50,000 newborns found that 30–40% of mothers with children, particularly those aged between 0 and 3 years, had experienced severe physical fatigue due to child-rearing [6–8]. The presence of young children in the home and the number of children may affect women’s physical and mental health [5, 9].

Factors associated with women’s health have been investigated in terms of socioeconomic conditions [5, 9]. However, the coping behaviors of mothers experiencing subjective symptoms remain unclear. The above-mentioned MHLW survey contained questions about coping behaviors for the most concerning symptoms, categorized according to whether mothers visited a doctor, used over-the-counter medication, or received no or some other form of treatment [1]; however, the results have not yet been published. Previous studies have shown that low socioeconomic status, the assumption of non-serious illness, and a low level of education are factors that contribute to patient delay in visiting a doctor [10–12]. In addition, Khan et al. reported that child-rearing mothers are reluctant to visit a doctor [13], although the details of this reluctance also remain unclear. To the best of our knowledge, no studies examining the coping behaviors of child-rearing mothers and their associated factors in terms of physical and mental subjective symptoms have been conducted.

Child-rearing mothers are reported to be at a lower risk of contracting serious life-threatening diseases than are the elderly. However, subjective symptoms or diseases can lead to greater hardships and higher medical costs, and can reduce social productivity resulting from work absenteeism [14]. Furthermore, a mother’s psychological condition can also have a major impact on that of her child [15, 16].

The present study sought to develop a conceptual framework for coping behaviors among child-rearing mothers experiencing physical and mental subjective symptoms, as well as the factors that influence these behaviors. This framework can be expected to serve as a fundamental resource for enabling child-rearing mothers to obtain the appropriate and required care when seeking medical treatment.

## Methods

Qualitative study. Study period: August 2013 to March 2014.

### Definition of terms

In the context of the present study, “any physical and mental subjective symptom” is used to refer to an “unwell or abnormal physical or mental state”; in other words, an unusual physical or mental state. Although “coping behavior” is generally used to describe the process of adapting to stress, in the present study, it is used to refer to “behavior to relieve unwell or abnormal physical or mental symptoms”. Therefore, it describes behavior intended to relieve not only mental, but also physical symptoms. Finally, “Fatigue” in the present study is used to refer to “a condition in which a person is physically or mentally tired.”

### Participants and setting

This study targeted those mothers who were currently raising children under 3 years of age in Japan and who had experienced physical and mental subjective symptoms since giving birth. This population was targeted due to the particularly significant fatigue involved in raising children up to the age of 3 years [6–8], and the fact that many children with stay-at-home mothers start attending kindergarten from the age of 3 years onwards. We planned to enroll a total of 20 to 30 mothers in accordance with previous studies [17, 18]. Participants were recruited via personal contacts, snowball sampling, and posters at a community center and nursery schools, and then contacted via telephone, e-mail, and face-to-face meetings. Theoretical sampling was performed with the aim of achieving theoretical saturation. Women who required regular medical exams or had difficulty communicating in Japanese were excluded from the study. Pregnant women were also excluded for the following reasons. First, pregnant women in Japan frequently go to the hospital (an average of about 14 visits) for health checkups, so they have numerous opportunities for periodic health checks. Second, pregnant women are sometimes in a state of physical and mental discomfort because of minor health problems such as morning sickness, backache, and constipation. Furthermore, they are at higher risk for illness than women who are not pregnant. These situations may also last longer in pregnant women, which could affect the content of interviews. However, we included data when the interviewees (non-pregnant mothers) told us about coping behaviors in a previous pregnancy period.

### Data collection

Data collection was undertaken by a female qualitative researcher (the first author), who was an assistant professor at the College of Nursing at the time of the study. All interview procedures were practiced several times in advance of the study. Next, semi-structured interviews were conducted in adherence with interview guidelines to clarify the participants’ coping behaviors when experiencing physical and mental subjective symptoms and associated factors. One 1-h interview was conducted with each of the participants and recorded using a digital voice recorder.

The interviews were conducted in the participants’ houses or office meeting rooms, or at the community center, university, or nursery schools. The location was chosen by the participants so that they could relax and feel confident that their confidentiality was protected. Upon the participants’ request, their children attended the interviews with them or stayed with babysitters in the same or a different room for the duration of the interviews.

### Analysis

Constant comparative method [17, 19–21]. First, field notes were written during and immediately after the interviews, and transcripts were created verbatim from recorded interviews and field notes. After carefully analyzing the transcripts, specific analytical themes were identified. Data were then extracted in contextual units based on the analytical theme, and concept names were generated after considering their definitions. An analytical worksheet composed of the name, definition, and specific examples of each concept, as well as a column designated for theoretical notes, was prepared to ensure that the analytical process was transparent and to check that the analysts had interpreted the interview responses correctly. Relationships between concepts were investigated and categorized, and the process was recorded in the theoretical notes column. Constant comparison of the data and concepts, the concepts themselves, and the concepts and categories was performed, and the relationships between concepts and categories and processes were graphically represented. Constant comparison was performed on both contrasting and similar examples. Theoretical saturation was defined as the point at which no more issues needed to be confirmed, even when new data were collected. The following tasks were performed to confirm theoretical saturation and ensure the validity of the data; 1) a study supervisor was appointed; 2) the data were examined by four qualitative researchers other than the study members; and 3) the interview respondents underwent member checking as a triangulation method. Member checking was performed using an anonymous mailing method to collect opinions about the interview results. The interviews and analyses were conducted in Japanese and then translated into English. A third party proficient in both Japanese and English then verified the accuracy of the translation. The results were presented in accordance with COREQ guidelines for reporting qualitative studies (Additional file 1) [22].

## Results

### Participants

None of the participants dropped out after being contacted by the interviewer. There were a total of 21 participants, 10 of who were stay-at-home mothers (indicated with an “S” in the interview transcripts) and 11 of whom were employed (indicated with a “W”). Nine of the employed mothers were professionals, six of whom were medical professionals (two nurses, one occupational health nurse, one physiotherapist, one pharmacist, and one acupuncturist). The median age of the mothers was 35 years (range: 24–42 years), and that of their youngest child was 19 months (range: 3–35 months). The median number of children was two (range: one to three). The interviewer had previous contact with three participants prior to study commencement.

### Concepts and categories

A total of 13 categories were created from 29 concepts identified from the analytical theme “*What do mothers do when raising children under 3 years of age while experiencing physical and mental subjective symptoms?*” (Table 1). Five of the 13 categories were core categories common among mothers, and eight were varied.

**Table 1:** Categories with Underlying Concepts, Definitions, and cited examples from the interview transcripts

^a^Categoriesy		Concepts		Concept definition and Cited examples^b, c^
1 (A)	Focus on the child even when feeling unwell	1	Focus on the child even when feeling unwell	Taking care of and focusing on the child even when in subjective symptoms
				*When I get a case of hives, I realize that it must be due to stress or fatigue, so I try to lie down as much as possible, when I can manage. I made an effort to get some rest, but when they’re little, they suddenly start crying because they want to be breastfed. It’s just not possible to have a proper rest. The baby always cries in the middle of the night. So after all that I don’t manage to get any rest, which causes my itching to flare up again. (S2)*
2 (A)	Hesitation in visiting a doctor	2	Reluctance to visit doctor with child	Increased reluctance to visit doctor due to mental and physical burden of visiting with child
				*When you’re not feeling well, you don’t want to take (your child) with you to the doctor. My children are very active, after all. If it’s just one child, then it probably wouldn’t be so bad. But if it’s all three children, well, it’s a hospital, so it’s not very hygienic. So I’m reluctant to take my children with me to see the doctor. I wouldn’t want them to get sick, so if I can avoid it, I’d rather not take them with me. (W5)*
		3	Hesitation to take medication while pregnant/breastfeeding	Hesitation to take medication while pregnant or breastfeeding even when in subjective symptoms due to concern that drug would be transmitted to baby via breastmilk
				*I used to get migraines, so I would go to the doctor and get some medication, but when you’re nursing, you can’t take medication. If you take medication, it will pass through your breast milk, so you just have to tolerate the pain. (S2)*
		4	Innate dislike of medication	Just dislike taking medication, even when in subjective symptoms
				*When (my headache) was really bad, I would take one tablet. Otherwise, if I think I can put up with the pain, then most of the time I just put up with it. I just refrain from taking medication. I don’t really like taking medication in the first place. I’m not a person who uses medication much. That’s why. (W10)*
		5	Reluctance to pay medical costs	Reluctance to pay medical costs for own treatment
				*When I was sick (with suspected sinusitis), I spent so much money going to the hospital because my family went so often. So I would try to endure because I didn’t have any money, and it would eventually heal on its own. (Omitted) At the time, I was working and I also had kids. But the main reason was because I had already spent my money on past hospital visits. Some months I would go to the hospital very often. My husband also went, so we decided to try and go without. (W8)*
		6	Reluctance to spend time visiting doctor	Time required to visit the doctor, including time spent visiting the hospital, waiting to see the doctor, and undergoing medical examinations, mean that mothers are reluctant in terms of physical hardship, considerable time spent looking after their child while at the hospital, and concern for their child while being looked after by others
				*(Reason for not visiting the doctor despite having migraines) First of all, going to the hospital costs money, and it also takes time. (W10)*
		7	Not wanting the hassle of searching for a hospital	Having no family doctor or regular hospital, bothersome to search for a hospital while in subjective symptoms
				*I still don’t have a primary care physician. Even before I became pregnant, I was generally healthy and didn’t visit the hospital much. Because I didn’t have a primary care physician, it was a hassle to try and find one...I think that’s why. (S4)*
3 (B)	Support difficult to use	8	Hesitation even to ask relatives	Hesitation to ask relatives such as mothers or husbands to look after their child or do housework in order to rest or visit the doctor, not wanting to impose, avoiding the physical effort required to make the trip, having concerns about the child’s reaction to being left with relatives
				*Even going back home (to my parent’s house) is tiring because we have to take the train, and wouldn’t even think of driving. (W1)*
		9	Burden of using temporary childcare	Using temporary childcare when in subjective symptoms brings physical burden of getting ready and traveling to the care facility, concern about the child’s condition and risk of infection while in care, mental burden of a lack of understanding on how to use the service, and financial burden of the cost of care
				*Even when I see a pamphlet for day care, I’m just not sure, (Omitted) So eventually I just give up on the day of using a day care service. It’s just not that easy to take my child there. (Omitted) Even if I used such a service, I don’t know the people there...so I don’t feel that it’s a type of support that I could use without any concerns. (W3)*
4 (B)	Housework even when feeling unwell	10	Housework even when feeling unwell	Performing housework even when feeling unwell
				*Yes, I do (housework even when in poor health). It basically makes no difference. Whether I’m sick or not, I still have to do it. (Omitted) When the norovirus was going around, I felt sick and quite nauseated, and I had to do all of the housework and disinfect everything. I was really exhausted. I was working while dry heaving. Then I vomited all over everything. All over the sheets and bedclothes. It took me so long to wash everything. (Omitted) I even made dinner wearing a surgical mask and gloves. (W11)*
5 (B)	Working even when feeling unwell	11	Working even when feeling unwell	Difficulty or inability to take time off work to rest or visit the doctor due to having work that precludes resting, using paid leave for their child, saving paid leave for future occasions, not wanting to worry about what colleagues think of them, not wanting a reduction in salary, not wanting to be rebuked for neglecting own health
				*(When my skin felt like I had a burn) I still had things to do as well as plenty of work at my workplace, so I honestly didn’t want to upset all that by taking annual leave to visit (the hospital). I don’t do that, and I also knew that it would be an imposition on others. (W6)*
6 (B)	No time to spend on myself	12	No time to spend on myself	Hesitation to devote time outside of work for working mothers to visiting the doctor or resting because hands are full with housework, taking care of children, finding time with the family
				*As soon as I finish work, I have to leave immediately to make sure that I can pick up my children by 7 p.m. After that, it’s already 7 p.m. and I want them to go to bed by 9 p.m., so once I feed and bathe them, there’s no time left. That’s why I don’t have time to do anything for myself. So I just do what I can to get the children to sleep. (Omitted) Then I do the housework, look after my husband, and prepare for the next day. Then the next morning comes and the battle starts all over again. (W7)*
7 (A)	Putting my own needs on hold	13	Putting my own needs on hold	Prioritizing child care and housework and/or work, putting own needs on hold, despite wanting to rest or visit the doctor
				*I frequently visit (the hospital) for my child but not for myself. I really haven’t been to the hospital for a personal matter. I’m always down the list in terms of priorities. I’m an adult so I can endure. (S5)*
8 (A)	Coping on my own as much as possible	14	Making an effort to rest	Attempting to rest and relieve symptoms as much as possible while child is sleeping or playing, or while someone is helping with housework or taking care of the child
				*I just try to get better by sleeping as much as possible. (Omitted) So at times like that when I’m exhausted, I just give my child some bread, and then I would just collapse. My child walks around eating bread and is covered in bread crumbs. She spills her tea as well. And I feel that I should clean it up, but I just can’t move, so on days like that I just leave the place in a mess. Then, if I just do nothing for a day, I usually feel better, then I can get some sleep. (S8)*
		15	Coping in my own way	Attempting to deal with symptoms in a variety of unique ways, such as massage or nutrition, not relying on rest or medication
				*I drink ginger tea every day, so when I’m starting to (catch a cold), I increase the amount of ginger. I put on a surgical mask and lie down. That’s what I do at first. (W9)*
		16	Misuse of prescription medication	Using previously-prescribed medication they have at home based on own judgment
				*I take my child to the hospital when she has a fever, and I would also take some of the cough medicine that was prescribed for my child (laughs). My child’s medicine. I thought if it was ok for children, then it would be fine for me. I heard that it works, so I thought I’d try some before I got sick. The cough syrup (laughs). But it really doesn’t work on adults. Herbal medicine also. Because prescription medication is expensive for adults. So I would use the medication that I received while pregnant. (S1)*
		17	Taking over-the-counter medication first	Using over-the-counter medications as a quick solution to alleviate symptoms rather than visiting the doctor and receiving prescription medication
				*When I think I have caught a cold, I want to go (to the hospital), but then I think about how I don’t want my child to get it, so I take over-the-counter (OTC) medication and try to put up with it. Yes. Going to the hospital (with my child) would be an unnecessary bother if he got a cold as well, so I decided to take OTC medication. (S6)*
		18	Discontinuing treatment on my own	Deciding to stop visiting the hospital or taking medication even when ongoing treatment is required
				*When I was pregnant, I went and saw a dentist. The doctor recommended me to treatment a tooth, but I didn’t do. I didn’t go there. I feel no problem, no problem. I have a carious tooth, but have not had a toothache so much yet. So, I finally decided not to go there. Once I was* free from pain after having a tooth hurts a little bit, I felt it was still ok. And then, I had medicine. I didn’t have a toothache so much. (S7)
9 (A)	Visiting the doctor with terrible symptoms	19	Visiting the doctor with terrible symptoms	Visiting the doctor when illness is so terrible that they cannot cope on their own and feel very ill, such as when symptoms worsen or persist, when feeling anxious, when needing some form of urgent medical treatment, etc.
				*I only visit the doctor when I’m very ill with a bad fever that just won’t go down and I have trouble breathing from coughing so much that I almost get pneumonia. (W4)*
10 (B)	Visiting the doctor early	20	Visiting the doctor early	Visiting the doctor early when mothers think they would pass on their illness to other family members or that their symptoms would worsen
				*I thought about visiting the doctor in the morning before my fever got worse. It was influenza season, so I thought that if I got sick, then all of children would get it too, so I suppose I just went to the doctor to get examined. (S8)*
11 (B)	Visiting the doctor while accompanying a family member	21	V Visiting the doctor while accompanying a family member	Consulting the doctor when accompanying child or family member, regardless of the extent of own symptoms
				*When my kids go to the dentist, I go with them and get a checkup and treatment at the same time. (W1)*
12 (B)	Predicting the cause, course, and treatment	22	Predicting the cause, course, and treatment	Before visiting the doctor, predicting the cause and course of their symptoms, examination results, doctor’s explanation of their condition, and their treatment regimen based on their own knowledge, experience, and information.
				*My most recent visit to the hospital was when I had influenza. Everyone in the family got sick one after the other and we knew it was the flu, so I went to the doctor to get Tamiflu. (W2)*
		23	Searching the Internet for starters	Gathering information on the cause of their symptoms, methods for coping, hospitals, etc., via the Internet
				*(When I had mastitis) I checked the Internet and it said that some doctors prescribe herbal medication containing arrowroot, so I just decided to take arrowroot. That worked, so I thought that’s all I have to do to fix it. (W11)*
		24	Searching for information in books	Gathering information on how to cope with their symptoms from books
				*When I got a mild case (vaginal candidiasis), I treated it myself once. I had the medication. Because my husband is a dermatologist. There’s that medication. So I read a book that said there was medication you could apply to the mucosa, so I used that and it got better. (S3)*
		25	Referring to the opinions of others	Discussing their physical condition with someone, draw on that person’s experiences and opinions
				*I had this constant pain in my stomach that just persisted for so long, and I was unable to eat because of the pain. Then I happened to bump into a childhood friend who was a nurse. (Omitted) She gave me advice and I had never had (a test), so she recommended that I just undergo the test once to put my mind at ease. So that was recommended by a friend. If it was just me, I probably wouldn’t have gone to get tested, but when a friend recommends it, I feel that I might as well go (to the hospital) once, and that was what motivated me to go. (S9)*
		26	Not gathering information when able to make a prediction	Failing to gather any information when capable of making their own decisions on the cause of their symptoms and ways to cope
				*I don’t particularly look up any information about my own health. When I contracted norovirus, it was obvious that I had the virus. With a cold, I usually get it from my kids, so I know it’s a cold. (S4)*
13 (B)	Use of support	27	Resting or visiting the doctor with help from relatives	Being able to rest and visit the doctor when in subjective symptoms because a family member takes care of their child, accompanies them to the clinic/hospital, or helps with the housework
				*When I go to the hospital, I usually have a high fever and can’t drive, so (my husband) takes time off work, or on some occasions, it happened to be a holiday, thankfully, all six times that I got sick. (S10)*
		28	Resting or visiting the doctor while the child was in day care	Mothers who take their child to day care can rest or visit the doctor while their child is under care
				*Yes, if I visit the doctor, it will be while my kids are at day care. (W9)*
		29	Managing the situation with the help of a friend	Managing to cope by resting, visiting the doctor, doing housework while a friend does the shopping or takes care of their child
				*(When my body felt heavy) my friend took care of the first child with playing in the pool at her house. I am really thankful for her who told me to lie down. (Omitted) If I didn’t lie down yesterday, I would not have made dinner. (S5)*

^a^(A) denotes core categories common among mothers. (B) denotes categories that varied among mothers

^b^Cited examples are shown in italics

^c^Each one cited example for each concept is indicated in this table. Moreover, additional cited examples r core concepts are indicated in the text

#### Relationships between categories (category names are shown in italics)

Even when they were experiencing subjective symptoms while raising a child under 3 years of age, mothers were “*Focused on the child even when feeling unwell*” and “*Hesitant to visit a doctor*”. Furthermore, some mothers did housework (“Housework even when feeling unwell”), went to work (“*Working even when feeling unwell*”), refused to make time for themselves (“*No time to spend on myself*”), or found it difficult to ask for help with child-rearing or housework (“*Difficult to ask for support*”).

The mothers were affected by these interacting circumstances, leading to a feeling of “*Putting my own needs on hold*”. This attitude influenced their coping behaviors while in poor health. As a result, mothers attempt to “Cope on their own as much as possible” and, at times, only visit a doctor after becoming seriously ill (“*Visiting the doctor with terrible symptoms*”). Furthermore, some mothers did “*Visit the doctor early*”, or “*Visit the doctor while accompanying a family member*”.

Their coping behaviors were influenced by “*Predicting the cause, course, and treatment*” of their symptoms and the “*Use of support*”. Mothers attempted to cope on their own when they felt capable, and visited the doctor only when they felt it was necessary. When receiving support, mothers were encouraged to rest and visit a doctor, and when they could not get any support, their visits to the doctor were hindered, leading to changes in their coping methods.

### Explanation of core categories common among mothers

#### Focused on the child even when feeling unwell

Mothers raising a child under 3 years of age were focused on their child even when experiencing physical and mental subjective symptoms. “(*When my child was hospitalized, I had flu symptoms*) *but I obviously couldn’t go to the hospital and leave my child, so I just slept with my child in her bed*” (W4). As shown in this response, the mother was focused on taking care of her child even when she suspected she had the flu herself. “*Each time I would have a fever of about 39°C or 40°C, so I would have to breast-feed the baby between these episodes of fever*. (Omitted) *Having to breast-feed my child. That was really tough*” (S10). This response demonstrated how mothers were focused on taking care of their child despite their unhealthiness.

#### Hesitation in visiting a doctor

This category describes the hesitation that mothers felt in visiting the doctor because of the reluctance to go together with their child, the hesitation to take medication, the reluctance to spend time or money at the doctor’s office, and the hassle of looking for a doctor. Most of the mothers for whom this category applied exhibited a reluctance to visit the doctor together with their child. Reasons for this reluctance included the concern that their child could contract an infectious disease, worry about bothering others if their child was unable to wait quietly during the appointment, and the physical burden of taking their child with them. Even when their child was not old enough to walk or crawl, or when they only had one child, mothers complained of a sense of burden. The longer the doctor’s appointment, the greater the reluctance showed by the mothers. “*It’s winter so I don’t want (my child) to catch a cold from someone*. (Omitted) *Even using a baby carrier is physically tiring. When my child cries, it’s a bother to others. When my child cries while we are at the hospital, I am considerate of others, so I tend to hesitate to visit the doctor*” (S2). “*My child has started crawling lately*. (Omitted) *It takes longer than usual to get anything done*. *For example, when paying the bill, I have to put the baby down like this, which is a bother*” (W8).

Mothers showing hesitancy to take medication while pregnant or breast-feeding were concerned that the medication would be transmitted to the fetus or the child via their breast milk. Symptoms that appeared in these situations included cold, fever, and gastroenteritis, and the medications involved included antibiotics, intestinal drugs, and antipyretics.

#### Putting my own needs on hold

Mothers put their own issues on hold even when experiencing physical and mental subjective symptoms due to the interaction of their child-centric lifestyle, their hesitation to visit the doctor, the difficulty of asking for support, and their work and/or housework commitments. This in turn influenced their coping behaviors. “*I frequently visit (the hospital) for my child, but not for myself. I really haven’t been to the hospital for a personal matter. I'm always down the list in terms of priorities*” (S5). “*I feel that I’m always putting myself last. When I work too hard. I wonder why that is. I’m afraid to ask for a day off*” (W3).

#### Coping on my own as much as possible

Because of their tendency to put their own issues on hold, mothers attempted to cope on their own as much as possible without visiting the doctor using four techniques. The first technique was resting. Mothers would focus on resting when taking their child to daycare or receiving support from relatives, or in other cases, would lie down while their child played beside them. “*I lie down on a mattress here (in the living room), then plead with my child to go to sleep and lay their toys on the floor, and then I lie down while they play*” (W6). The second technique was a unique approach that did not involve rest or medication. “*(When I suspect that I have mastitis) I massage myself most of the time to relieve the breast engorgement, but when I can’t relieve it on my own (I visit the doctor)*” (S9). The third technique was to take medication at home from past prescriptions, including antipyretics, antibiotics, and other antifungal drugs. “*I saved the (antipyretic) medication that I had previously been prescribed (by the hospital), which was safe for nursing mothers, so I took it when I suddenly came down with a high fever*” (W2). The fourth technique was to use over-the-counter medication. “*I thought that going to the hospital (with my child) would be an unnecessary bother if he just caught a cold, so I decided to take over-the-counter medication*” (S6). In addition to these four coping methods, some mothers decided to discontinue treatment and cope on their own, even when medical care was required. “*I had wanted to go to the dentist but my husband wouldn’t or couldn’t look after our child, so I was reluctant to go. Then our second child was born, so I was too busy to go (to the dentist), so my teeth have been like this (shows untreated teeth) for about a year now. They filed them down as much as possible and removed the gum tissue, and they are still in this condition today*” (S1).

#### Visiting the doctor with terrible symptoms

Many of the mothers did in fact visit the doctor when they felt terrible. “*(When my nasal congestion improved) I couldn’t smell anything. Although my nose wasn’t blocked, even when I took the cap off a bottle of perfume and tried to smell it, I couldn’t. I was concerned so I took the cap off the gasoline tank and tried to smell it, but still couldn’t smell anything. That had me worried. I couldn’t taste what I ate, and I had only had a slight sensation of sweet, spicy, or sour, so I was quite concerned (and I visited the doctor)*” (S3). One mother noted her experience with the threat of premature delivery in a previous pregnancy period. “*I put (my own needs) on hold. My child is far and away my top priority, and everything else is irrelevant. Even when that (hospitalization due to threatened premature delivery) occurred, it was no good. I definitely pushed myself too hard.* (Omitted) *So I probably could have avoided going to hospital if I had been more careful, but I can’t help myself*” (W11).

## Discussion

In the present study, mothers tried to cope on their own rather than visiting a clinic or hospital when experiencing physical and mental subjective symptoms. As a result, most of the mothers only visited the doctor after their symptoms had worsened. One factor that had a significant demonstrable effect on coping behaviors during times of subjective symptoms was the mother’s feeling of putting her own needs on hold. Factors that influenced this feeling included a child-centric lifestyle, hesitation in visiting a doctor, work and/or housework, and the difficulty of asking for support.

This feeling of putting one’s needs on hold was also affected by a reluctance to visit the doctor due to associated time and medical costs, as well as work and housework, so it is possible that this feeling is not exclusive to child-rearing women. However, in the present study, this feeling was largely influenced by factors related to their children. With this in mind, there are four cultural background factors in Japan that make child-rearing mothers tend to put their own needs on hold. The first is that in Japan, the mother is predominantly responsible for raising the children and doing the housework. A government survey has shown that in approximately 70% of married couples aged 20–49 years, the wife is responsible for at least 70% of child-rearing and household tasks, even when both the husband and wife are employed full-time [23]. In Japan, the roles of raising the child and doing the housework tend to be assigned to women, so it is conceivable that they put their own needs on hold in order to fulfill these roles. The second cultural factor is that much of the support for child-rearing comes from relatives [24]. Married couples who do not receive any support from relatives lack the means to prioritize their own needs [24]. A survey by the Cabinet Office revealed that 85% of couples rely on direct support from their relatives in the form of childcare and shopping, whereas 27% utilize public support services such as temporary childcare services, and around 10% use paid support services such as babysitters [25]. The third factor is Japan’s work environment, which makes it difficult to take paid leave. A survey of Japanese working conditions by the MHLW showed that only around 10% of companies allow employees to use their paid annual leave on an hourly basis [26], which could explain why working mothers are hesitant to visit the doctor, even when it would only take a few hours. Children under the age of 3 years are also susceptible to infections, so mothers tend to use their paid holidays for their child rather than for themselves. The fourth factor is the cultural trait of putting one’s family first, even when it involves self-sacrifice. In a survey by the Cabinet Office, approximately 70% of respondents claimed that they felt obliged to make sacrifices for their family rather than prioritizing their own lifestyle [27].

A total of seven factors were identified as having contributed to mothers’ “Hesitation in visiting a doctor”. Two of these factors took the form of a hitherto unidentified reluctance to visit the doctor when raising a child. The first was reluctance in visiting the doctor together with one’s child. Mothers were worried about causing a disturbance to others, as most children under the age of 3 years find it difficult to wait quietly. Furthermore, the reluctance of mothers to visit the doctor with their child was grounded in a sense of physical burden and the risk of infection to their child. This finding suggests that if mothers could access public and private support services to meet their needs, they would be less concerned about disturbing others or the risk of their child contracting an infection at the clinic or hospital, which would in turn reduce their hesitation in visiting a doctor. The second factor was the reluctance to take medication while pregnant or breast-feeding, particularly while breast-feeding, even though there were oral medications that the mother could have taken safely to alleviate her symptoms [28, 29]. The hesitation of mothers to take medication may have been based on their own general knowledge as well as professional medical advice about medication while breast-feeding. Providing information about medication that can be taken during pregnancy and breast-feeding to medical professionals and women who are pregnant or breast-feeding could help relieve the hesitation that mothers have towards taking medication.

Under the concept “A unique approach that did not involve either rest or medication”, one participant performed self-care (breast massage) when she suspected mastitis. There is no evidence regarding the efficacy of breast massage for mastitis; however, in Japan, traditionally, and even at present, midwives perform breast massage on breastfeeding mothers, and sometimes teach them simple breast massage as a self-care method. Therefore, we considered that mothers performed self-care through the breast massage method recommended by midwives.

Methods of gathering information for “*Predicting the cause, course, and treatment*” of their subjective symptoms consisted of using external information sources via the Internet. This finding is consistent with those from a study by Bouche et al. reporting that mothers obtained medical and health information via the Internet [30]. A survey by Japan’s Ministry of Internal Affairs and Communications found that the Internet penetration rate among individuals aged 20–39 years was 98% to 99%, and that smartphone use was approximately 90% [31]; women of infant-rearing age had a high rate of Internet use. Despite the issues of health literacy and the accuracy of information regarding Internet use, child-rearing women tend to use the Internet as a means of obtaining health information. Providing accurate information in response to queries by mothers could be useful in predicting the cause, course, and treatment of their poor health and encourage them to adopt proper self-coping behaviors and visit the doctor.

The symptoms experienced by mothers who participated in this study ranged from chronic conditions such as arthritis, migraine, and toothache, to acute conditions such as influenza, acute gastroenteritis, mastitis and external injury, as well as hospitalization due to threatened premature delivery. The seriousness of symptoms may have affected their coping behaviors. However, the study participants (who were all raising children under the age of 3 years) tended to focus on their child even when experiencing physical and mental subjective symptoms and feeling unwell, and as a result, were more likely to put their own needs on hold to the extent that they tended to refrain from visiting a doctor until they were seriously ill.

This study did have two noticeable limitations. First, we performed sampling in an attempt to prevent bias regarding the participants’ occupations and number of children. However, due in part to the effect of personal contacts and snowball sampling, six of the 11 participants who were employed turned out to be medical professionals. Although their medical occupations varied from nurses to acupuncturists and physiotherapists, the medical knowledge and experience of these participants could have affected how they predicted the cause, course, and treatment of their subjective symptoms, as well as the extent to which they were capable of self-coping and putting off a visit to the doctor. Therefore, in this study, we placed an emphasis on confirming theoretical saturation, including the results of stay-at-home mothers and those with other occupations. The second limitation was that the main investigator (the first author), who conducted the interviews and analysis, had interacted with child-rearing mothers in her capacity as a midwife, and was herself a mother of young children; therefore, her past experiences and attitudes may have influenced the study outcomes. We therefore used a constant comparative methodology, ensured that the study was supervised, requested a review of the study results by qualitative researchers other than the study members, and performed triangulation-based checking of study members in an attempt to preclude any arbitrary analyses. We also used an analytical worksheet to clarify the analytical process and enable valid interpretation of the results based on the data. Although the first author viewed the behavior of “putting one’s own needs on hold” as a matter of course rather than as a hypothetical idea, this result was derived through analysis of the data.

In Japan, the number of working mothers is increasing [32, 33], and the childbearing age is rising (28% of all women who give birth are 35 years of age or older) [34] similar to many developed countries. Working mothers experience a greater physical and mental burdens such as higher norepinephrine levels after work compared with others [9, 35–37]. Moreover, a tendency to feel fatigue increases as the childbearing age rises. Therefore, greater attention needs to be paid to mothers’ subjective symptoms and associated coping behaviors, as well as the contributing factors. Through this study, we have developed a conceptual model on the coping behaviors of mothers when experiencing subjective symptoms and the contributing factors. It is our hope that this model will help foster an environment in which mothers can visit a doctor whenever they wish by focusing attention on improvements to maternal and child health policies as well as healthcare and occupational settings.

## Conclusions

The study findings showed that mothers with children under the age of 3 years in Japan put their own needs on hold when experiencing physical and mental subjective symptoms due not only to common factors, but also to child-rearing-related factors. As a result, mothers attempt to cope on their own as much as possible and, at times, only visit a doctor after becoming seriously ill.

## Additional file

Additional file 1: Consolidated criteria for reporting qualitative studies (COREQ): 32-item checklist [22]. This data is to ensure our manuscript adheres to COREQ guidelines for reporting qualitative studies. (DOCX 128 kb)

## Abbreviation

MHLW: Ministry of Health, Labor and Welfare
