# Supplementary material for: A qualitative study on coping behaviors and influencing factors among mothers in Japan raising children under three years old while experiencing physical and mental subjective symptoms
Source: BMC Womens Health. 2018 Jan 10;18:14. doi: 10.1186/s12905-017-0494-8 (PMC5764027; doi:10.1186/s12905-017-0494-8)
Supplement: Additional file 1: — Consolidated criteria for reporting qualitative studies (COREQ): 32-item checklist [22]. This data is to ensure our manuscript adheres to COREQ guidelines for reporting qualitative studies. (DOCX 128 kb) [file 12905_2017_494_MOESM1_ESM.docx]

**Consolidated criteria for reporting qualitative studies (COREQ): 32-item checklist**

|  | **Item** | **Guide questions/description** |  |
| --- | --- | --- | --- |
| **Domain 1: Research team and reflexivity** | | |  |
| **Personal Characteristics** | |  |  |
| 1. | Interviewer/facilitator | Which author/s conducted the interview or focus group? | The first author.  (Page 3, the first column) |
| 2. | Credentials | What were the researcher's credentials? *E.g. PhD, MD* | We wrote the researcher's credentials in the title page as followings;  MK (MPH), KM (PhD), and TN (MD, PhD). |
| 3. | Occupation | What was their occupation at the time of the study? | An assistant professor at the College of Nursing at the time of the study.  (Page 3, the first column) |
| 4. | Gender | Was the researcher male or female? | Data collection was undertaken by a female qualitative researcher.  (Page 3, the first column) |
| 5. | Experience and training | What experience or training did the researcher have? | All interview procedures were practiced several times in advance of the study. (Page 3, the first column) |
| **Relationship with participants** | | |  |
| 6. | Relationship established | Was a relationship established prior to study commencement? | The interviewer had previous contact with three participants prior to study commencement.  (Page 3, the second column) |
| 7. | Participant knowledge of the interviewer | What did the participants know about the researcher? e*.g. personal goals, reasons for doing the research* | *Ethics approval and consent to participate* in Declarations.  Prior to enrollment and again before commencing the interviews, all participants were informed about the purpose and methods of the study.  (Page 10, the second column) |
| 8. | Interviewer characteristics | What characteristics were reported about the interviewer/facilitator? e.g. *Bias, assumptions, reasons and interests in the research topic* | The second limitation was that the main investigator (the first author), who conducted the interviews and analysis, had interacted with child-rearing mothers in her capacity as a midwife, and was herself a mother of young children; therefore, her past experiences and attitudes may have influenced the study outcomes. We therefore used a constant comparative methodology, ensured that the study was supervised, requested a review of the study results by qualitative researchers other than the study members, and performed triangulation-based checking of study members in an attempt to preclude any arbitrary analyses. We also used an analytical worksheet to clarify the analytical process and enable valid interpretation of the results based on the data. Although the first author viewed the behavior of “putting one’s own needs on hold” as a matter of course rather than as a hypothetical idea, this result was derived through analysis of the data.  (Page 10, the first column) |
| **Domain 2: study design** | | |  |
| **Theoretical framework** | | |  |
| 9. | Methodological orientation and Theory | What methodological orientation was stated to underpin the study? *e.g. grounded theory, discourse analysis,* | Constant comparative method.  (Page 3, the first column) |
| **Participant selection** | |  |  |
| 10. | Sampling | How were participants selected? *e.g. purposive, convenience, consecutive, snowball* | Participants were recruited via personal contacts, snowball sampling, and posters at a community center and nursery schools.  (Page 2, the second column) |
| 11. | Method of approach | How were participants approached? e*.g. face-to-face, telephone, mail, email* | Participants were contacted via telephone, e-mail, and face-to-face meetings.  (Page 2, the second column) |
| 12. | Sample size | How many participants were in the study? | There were a total of 21 participants.  (Page 3, the second column) |
| 13. | Non-participation | How many people refused to participate or dropped out? Reasons? | None of the participants dropped out after being contacted by the interviewer.  (Page 3, the second column) |
| **Setting** | |  |  |
| 14. | Setting of data collection | Where was the data collected? e*.g. home, clinic, workplace* | The participants’ houses or office meeting rooms, or at the community center, university, or nursery schools. The location was chosen by the participants so that they could relax and feel confident that their confidentiality was protected.  (Page 3, the first column) |
| 15. | Presence of non-participants | Was anyone else present besides the participants and researchers? | Upon the participants’ request, their children attended the interviews with them or stayed with babysitters in the same or a different room for the duration of the interviews.  (Page 3, the first column) |
| 16. | Description of sample | What are the important characteristics of the sample? *e.g. demographic data, date* | This study targeted those mothers who were currently raising children under 3 years of age in Japan and who had experienced physical and mental subjective symptoms since giving birth. Women who required regular medical exams or had difficulty communicating in Japanese were excluded from the study. Pregnant women were also excluded.  (Page 2, the second column) |
| **Data collection** | |  |  |
| 17. | Interview guide | Were questions, prompts, guides provided by the authors? Was it pilot tested? | Semi-structured interviews were conducted in adherence with interview guidelines to clarify the participants’ coping behaviors when experiencing physical and mental subjective symptoms and associated factors.  (Page 3, the first column) |
| 18. | Repeat interviews | Were repeat interviews carried out? If yes, how many? | One.  (Page 3, the first column) |
| 19. | Audio/visual recording | Did the research use audio or visual recording to collect the data? | A digital voice recorder.  (Page 3, the first column) |
| 20. | Field notes | Were field notes made during and/or after the interview or focus group? | Field notes were written during and immediately after the interviews.  (Page 3, the first column) |
| 21. | Duration | What was the duration of the interviews or focus group? | 1-hour.  (Page 3, the first column) |
| 22. | Data saturation | Was data saturation discussed? | Theoretical saturation was defined as the point at which no more issues needed to be confirmed, even when new data were collected. The following tasks were performed to confirm theoretical saturation and ensure the validity of the data; 1) a study supervisor was appointed; 2) the data were examined by four qualitative researchers other than the study members; and 3) the interview respondents underwent member checking as a triangulation method. Member checking was performed using an anonymous mailing method to collect opinions about the interview results.  (Page 3, the first column) |
| 23. | Transcripts returned | Were transcripts returned to participants for comment and/or correction? | No. We don’t return it to participants. |
| **Domain 3: analysis and findings** | | |  |
| **Data analysis** | |  |  |
| 24. | Number of data coders | How many data coders coded the data? | A total of 13 categories were created from 29 concepts.  (Page 3, the second column) |
| 25. | Description of the coding tree | Did authors provide a description of the coding tree? | *Relationships between categories* and *Explanation of core categories common among mothers* in the Results, and Table 1.  (Pages 3-8) |
| 26. | Derivation of themes | Were themes identified in advance or derived from the data? | After carefully analyzing the transcripts, specific analytical themes were identified.  (Page 3, the first column) |
| 27. | Software | What software, if applicable, was used to manage the data? | No. We didn’t use it. |
| 28. | Participant checking | Did participants provide feedback on the findings? | The interview respondents underwent member checking as a triangulation method. Member checking was performed using an anonymous mailing method to collect opinions about the interview results.  (Page 3) |
| **Reporting** | |  |  |
| 29. | Quotations presented | Were participant quotations presented to illustrate the themes / findings? Was each quotation identified? e*.g. participant number* | *Explanation of core categories common among mothers* in the Results and Table 1.  (Pages 4-8) |
| 30. | Data and findings consistent | Was there consistency between the data presented and the findings? | *Relationships between categories* and *Explanation of core categories common among mothers* in the Results, and Table 1.  (Pages 3-8) |
| 31. | Clarity of major themes | Were major themes clearly presented in the findings? | *Explanation of core categories common among mothers* in the Results and Table 1.  (Pages 4-8) |
| 32. | Clarity of minor themes | Is there a description of diverse cases or discussion of minor themes? | *Relationships between categories* in the Results and Table 1.  (Pages 3-7) |

Allison Tong, Peter Sainsbury, Jonathan Craig. Consolidated criteria for reporting qualitative research (COREQ): a 32-item checklist for interviews and focus groups. International Journal for Quality in Health Care*.* 2007;19(6):349-357; doi:10.1093/intqhc/mzm042
